# Packaging and Optimization of a Capacitive Biosensor and Its Readout Circuit

**DOI:** 10.3390/s23020765

**Published:** 2023-01-09

**Authors:** Antonios Georgas, Lampros Nestoras, Aris Ioannis Kanaris, Spyridon Angelopoulos, Angelo Ferraro, Evangelos Hristoforou

**Affiliations:** School of Electrical and Computer Engineering, National Technical University of Athens, Zografou Campus 9, Iroon Polytechniou Str., 15780 Zografou, Greece

**Keywords:** 3D printing, ORDYL SY 300, interdigitated electrodes, electronic packaging, biosensor optimization

## Abstract

In pipeline production, there is a considerable distance between the moment when the operation principle of a biosensor will be verified in the laboratory until the moment when it can be used in real conditions. This distance is often covered by an optimization and packaging process. This article described the packaging and optimization of a SARS-CoV-2 biosensor, as well as the packaging of its electronic readout circuit. The biosensor was packed with a photosensitive tape, which forms a protective layer and is patterned in a way to form a well in the sensing area. The well is meant to limit the liquid diffusion, thereby reducing the measurement error. Subsequently, a connector between the biosensor and its readout circuit was designed and 3D-printed, ensuring the continuous and easy reading of the biosensor. In the last step, a three-dimensional case was designed and printed, thus protecting the circuit from any damage, and allowing its operation in real conditions.

## 1. Introduction

As biosensor systems are used in more and more areas of public health, food, and environmental technology as well as in the general global economy, the need to optimize them for use in real conditions is ever-increasing. Capacitive biosensors are one of the most popular types, as they are considered highly sensitive and can provide results in a short time [1]. The basic operational principle in most capacitive biosensors is the following: (i) a capacitive transducer is selected; (ii) and its surface is coated with a layer containing a bioreceptor able to bind the target analyte(s). Then, the sample containing the target analyte is placed on top of the biosensor surface and the binding among the bioreceptor and analyte results in a change in the measured capacitance [2].

However, there are certain challenges that need to be overcome regarding capacitive biosensors [3]. A major challenge in the development of capacitive biosensors, especially in the design of their readout circuits, is the treatment of noise interference [4,5] and, consequently, the issues that arise in relation to measurements stability and repeatability. Sensor stability is related to the system’s ability to maintain excellent performance independently of external factors, while repeatability is related to the ability of the sensor to provide the same results in the same operating conditions.

Both stability and repeatability may be affected by the sample’s diffusion over the biosensor surface, and consequently the analyte. When placing a drop of any liquid specimen over the biosensor surface, for example, the liquid may start spreading to a larger part of the surface, leading to an increase in the dielectric constant and consequently to an increase in the measured capacitance. Therefore, it is important to control the liquid sample diffusion on top of sensor surface in order for it to be confined in the selected area [6].

Furthermore, in real conditions it is critical to protect the sensor’s sensitive parts (bioreceptor). Since both biosensors and electronics are designed to be used in micro- or nano-scale [7], they can be easily damaged. A common simple, robust and low cost solution is to use photosensitive polymers, which form a bond and, at the same time, seal the layers at the biosensor’s surface [8,9].

Biosensors are usually accompanied by an electronic readout circuit, which is mainly used to amplify the measured signal and noise compensation as well. The development of integrated circuits (ICs) and microelectromechanical systems (MEMS) has made great strides in every aspect of technology and especially in industry. In order to be able to use them, it is necessary that these circuits are properly packaged. Their packaging usually provides a variety of functions [10,11,12,13], such as protection from environmental factors, as any chemical changes may reduce the efficiency of the circuit, as well as mechanical strength and heat management both to increase the reliability and service life of the circuit and to prevent material damage [14]. The circuit also needs to be interconnected with the packaging to enable its interaction with a user, another circuit, or a larger system.

Modern packaging techniques must be defined at the beginning of the product or circuit design to match the entire package during the assembly process, but without compromising the performance of the device. Also, it is usually required to order a large number of prototypes made from many different materials, thus increasing the cost of manufacturing. 3D printing technology simplifies this process [15]. Spraying techniques have already been used to create 3D ICs [16] as well as to encapsulate MEMS devices [17,18].

In our previous work, we developed a capacitive biosensor for SARS-CoV-2 detection in real time [19]. Afterwards, we developed a precise biosensor readout circuit, thus turning the biosensor into a Point of Care (PoC) diagnosing device [20,21]. In this study we used an acrylic-based dry film, ORDYL SY 300 [8,22,23], to package the SARS-CoV-2 biosensor, addressing both the issue of biosensor bonding and sealing, as well as to control liquid sample diffusion on the biosensor’s surface. More specifically, we realized a protective layer on the biosensor surface to avoid damage related to storage and transport. Additionally, we patterned wells by using UV lithography on the biosensor’s sensitive area, where the liquid sample will be placed. As a result, the liquid remains confined, mitigating the noise introduced by liquid sample diffusion. Finally, we used 3D Printing technology to develop the connector between the biosensor and the electronic readout circuit, as well as the packaging of the readout circuit.

## 2. Materials and Methods

### 2.1. Sensor Coating with ORDYL SY 300

**Figure 1:**
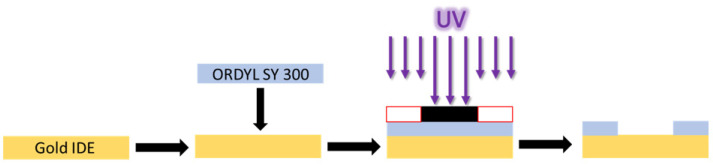
Coating and patterning ORDYL SY 300 at gold IDE surface.

Gold interdigitated electrodes (IDEs) were purchased from DropSens (Asturias, Spain), cat. N.: PW-IDEAU50. Each IDE has a finger width and spacing of 50 μm, with a total number of 70 fingers, a total electrode length of 7 mm, and electrode surface area of 8.45 mm^2^. Initially, the IDEs were immersed in an acetone solution, then in 100% isopropanol and finally, they were washed with distilled water. Chemicals were purchased from Sigma-Aldrich (St. Louis, CA, USA). For sensor coating, it was chosen to use dry resin, specifically ORDYL SY 300, which is a permanent dry film with a very high possible aspect ratio and excellent high thermal and chemical stability. The experimental procedure is shown in Figure 1; The IDEs were placed in a hot plate at 110 °C. An ORDYL SY 300 piece, slightly larger than the IDE sensitive area, was cut, ORDYL protective liner was removed from the one side and the tape was coated at the IDE surface. A photolithography mask was designed and printed. The sample was exposed at UV for 48 s (0.8 min The white dark areas of the mask protected the photosensitive layer from UV, whereas the dark transparent areas were exposed. The ORDYL protective liner was removed from the other side, revealing its photosensitive surface. Then, it was immersed in its developer solution, in an ultrasonic bath, for another 5 min. As a result, the ORDYL SY 300, being negative photoresist, was removed from the areas protected from the UV exposure. After the developing process, the sample was immersed again in 100% isopropanol solution and washed with distilled water. At the end, it was placed at a hot plate at 120 °C for 45 min, in order to harden the ORDYL layer. The thickness of the ORDYL layer before the post baking process was 90 μm.

### 2.2. Connector Design

Due to the small size of the measuring capacitor’s electrodes, a connector had to be made in order to obtain readings without damaging the capacitor. Solutions such as soldering connectors on the surface had to be avoided and replaced by mechanical means to keep the capacitor in place and provide constant electrical connection with the readout circuit.

**Figure 2:**
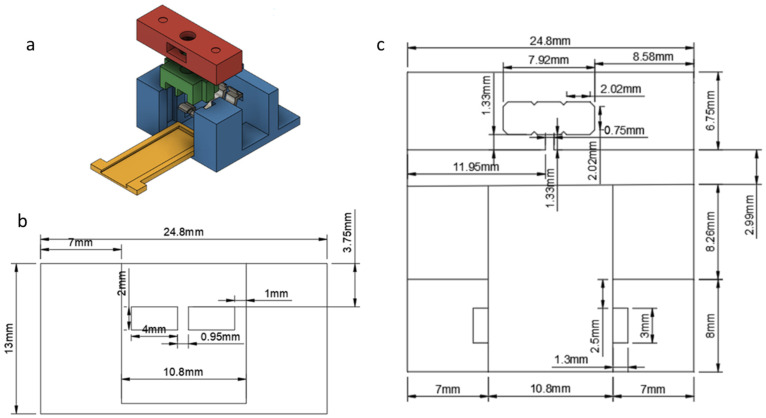
Design of the connector hosting the biosensor: (**a**) schematic; (**b**) front view; and (**c**) top view.

For the electrical connection, bent strips of stainless steel were used, as shown in Figure 2. Part A (Red) was fixed on the base (blue) with metallic rods, having holes for a screw and nut mechanism. Turning the screw tightens Part B (green), which in turn tightens the metallic strips, ensuring the electrical contact with the capacitor’s electrodes.

A recessed case (yellow) was also designed, so that the capacitor would be placed and remain during the measurements. On the back side of the connector the metallic strips were connected with male pins which provide connectivity with the readout device. The exact dimensions of the connector are shown in Figure 2b (front view) and Figure 2c (top view).

### 2.3. Packaging Design of Readout Circuit

The design of the packaging for the readout circuit had to provide connectivity for the Input/Output (I/O) components of the board. The most important of all was the ability for the circuit and the capacitor connector to form a unified unit. With that in mind, a case was designed including the proper holes and cutouts for the I/O components (Connector, LCD, Buttons etc.) so that the board was held safely in place.

**Figure 3:**
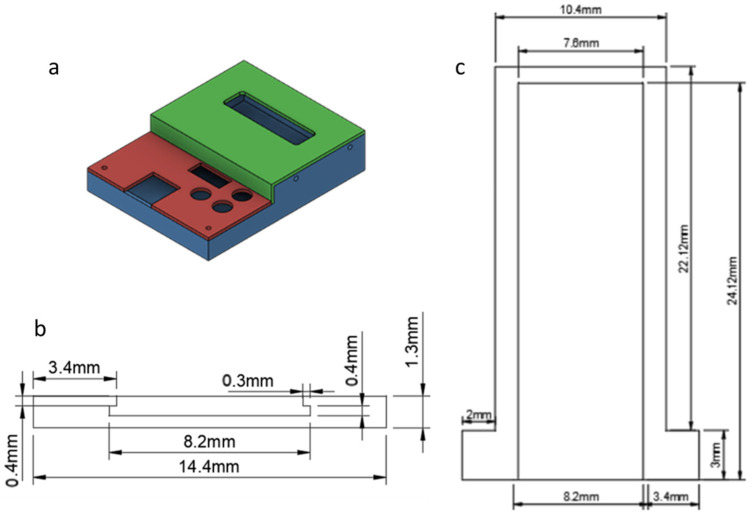
Readout circuit packaging design: (**a**) schematic; base (blue); bottom lid (red); top lid (green); (**b**) front view; and (**c**) top view.

The packaging consisted of three parts, shown in Figure 3:

- The base (blue) on which the board was mounted;
- Bottom lid (red) for the lower part of the readout circuit;
- Top lid (green) for the upper part of the readout circuit.

All the parts were designed in Fusion360^®^, sliced in Simplify3D^®^ and then 3D-printed on an Anycubic i3 Mega-S FDM 3D printer using 1.75 mm PLA filament of the same brand. The exact dimensions of the package are shown in Figure 3b (front view) and Figure 3c (top view).

### 2.4. Data Processing and Evaluation

For better results display, the capacitance was normalized. Therefore, the capacitance values presented in the “Results and Discussion” section are divided by the maximum capacitance value of each experiment.

To compare the performance of the coated biosensors against the biosensors without patterned wells, in both cases the standard deviation was calculated according to Equation (1):

(1) $$\sigma =\sqrt{\frac{1}{n-1}\overset{n}{\underset{1}{\sum }}{\left(x_{i}-\bar{x}\right)}^{2}}$$

where *n* corresponds to the number of measurements, $\bar{x}$ corresponds to the average value and *x_i_* corresponds to each measurement.

For the characterization of the coated ORDYL SY 300 layer, an optical profilometer was used (TMS 1200, Polytec Inc., Mooresville, NC, USA) in order to measure the post-baking thickness and to evaluate the layer homogeneity.

## 3. Results and Discussion

### 3.1. Photosensitive Layer on Interdigitated Surface

**Figure 4:**
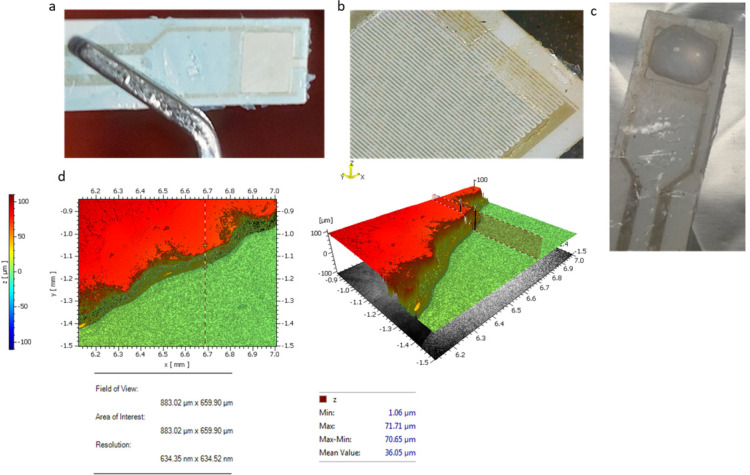
Patterning square-shaped wells on capacitor sensitive area: (**a**) image of the patterned well; (**b**) image of the well in 20× magnification; (**c**) a droplet is placed at the well; and (**d**) ORDYL thickness measured with optical profilometer.

Following the procedure described in Section 2.1, an ORDYL SY 300 layer was coated on the interdigitated capacitor surface. The aim of the coating was to achieve good control of the sample liquid diffusion over the sensory capacitor area, as well as to create a protective layer on the rest of the capacitor surface, in order to avoid any damage. As illustrated in Figure 4a, an ORDYL layer was coated at the capacitor surface. Then, a square-shaped well was patterned at the biosensor’s sensitive area where the liquid sample was placed. A digital microscope (BRESSER, Rhede, Germany) was used for the optical characterization of the coating with a 20X magnification. The magnified picture is shown in Figure 4b. A liquid drop was placed at the well square area, as illustrated in Figure 4c. Figure 4d shows the post-baking thickness of the ORDYL layer, which, as expected, was reduced to about 70 μm after the baking process from the initial thickness of 90 μm.

**Figure 5:**
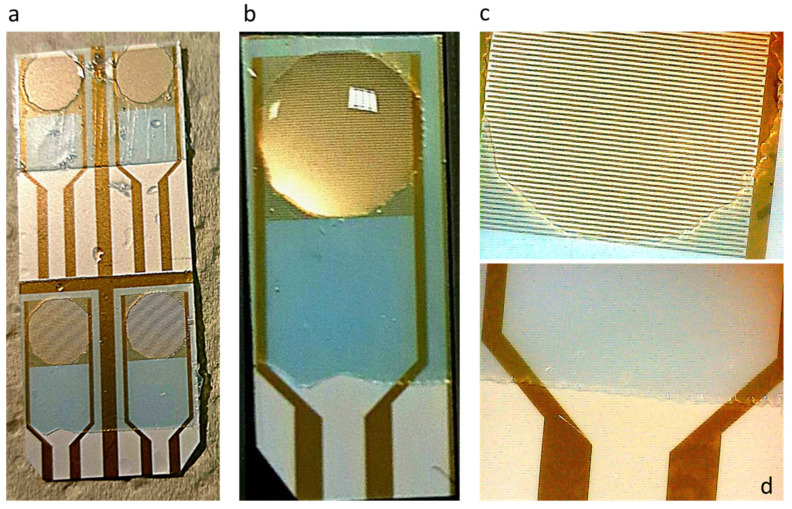
Patterning circular wells on capacitor sensitive area: (**a**) wells patterned on a set of capacitors; (**b**) a droplet is placed at the well; (**c**) image of the well in 20× magnification; and (**d**) ORDYL layer does not cover the capacitor pads.

Due to superficial tension forces, a problem with the square geometry was noticed, as the liquid was not spreading evenly at the well corners. For that reason, a circular geometry was tested. An ORDYL layer was realized on top of a group of four capacitors. After UV exposure, circular wells were patterned at the sensitive area, as shown in Figure 5a. ORDYL layer was not deposited on the capacitor pads, so that they could be connected with the readout device, as shown in Figure 5d (20× magnification). After ORDYL treatment, the capacitors were separated and used one at a time. With this geometry, the liquid was spreading perfectly in the well, as shown in Figure 5b,c (20× magnification). ORDYL SY 300 was selected for the sensor coating, because it is biocompatible, it remains permanently at the surface after coating, and it is very easy to use in comparison to other dry resins.

**Figure 6:**
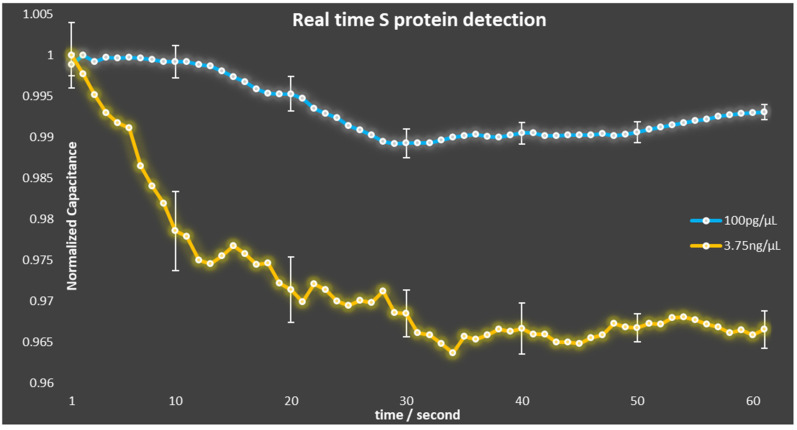
Normalized capacitance change over time due to the binding of S protein.

After the well geometry configuration was optimized, the modified capacitors were utilized to realize the SARS-CoV-2 biosensors. The biosensors were prepared following the procedure described in our previous work [19] and their response to different concentrations of viral S protein was monitored, as shown in Figure 6. For each S protein concentration, a total of three experiments were conducted. The lines shown in Figure 6 correspond to the average measurement for each concentration, whereas the error bars correspond to the standard error of the measurements. The main purpose of these measurements was to validate that the capacitors were not damaged and were still functional after the modification process. In addition, the standard deviation of the measurements using the biosensors with patterned wells was improved compared to the measurements using biosensors without wells. More specifically, according to Equation (1), the standard deviation of the first case was calculated equal to σ = 0.004950, whereas the standard deviation of the latter was calculated equal to σ = 0.065194.

### 3.2. Connector Development

The connector consisted of four total parts to be printed:

- Packaging base;
- Driver;
- Fixing cap;
- Sensor case.

**Figure 7:**
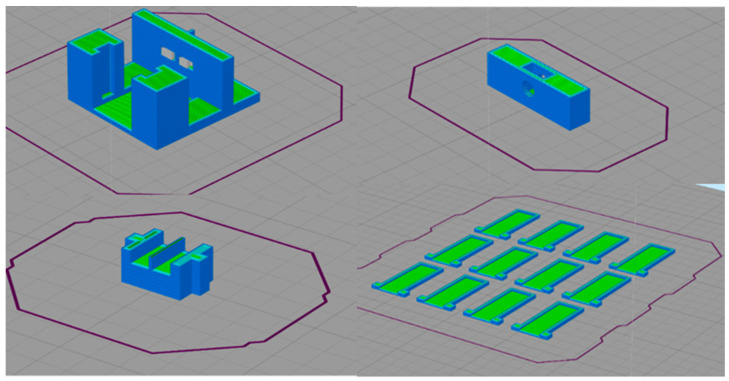
Connector slicing process.

Figure 7 shows the process of slicing the parts that make up the connector. Where necessary, a support material was placed and optimal print settings, shown in Table 1, were selected.

**Figure 8:**
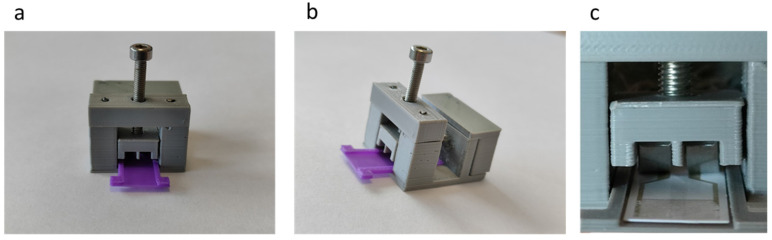
The 3D printed connector: (**a**) front view; (**b**) side view; and (**c**) the connection of the conductive plates to the sensor.

Next, the individual pieces of the connector were assembled, as shown in Figure 8a (front view) and Figure 8b (side view). Since the plates are made of stainless steel, welding them to the interface pins was not possible through conventional welding. For this reason, the back of the plates was wrapped with copper wire and then soldered. Also shown in Figure 8c is the position of the plates in relation to the electrodes, which was the optimum, since the plates touch the entire area of the electrodes. Finally, the conductive parts were insulated with silicone. Connector assembly was completed by placing the retaining screw and nut on the top of the package, which was braced with small diameter metal rods. The sensor case, shown with purple, was also manufactured with 3D printing.

### 3.3. Electronic Circuit Packaging

The electronic circuit packaging consists of three parts:

- The base of the package;
- The lid of the upper part;
- The bottom lid.

Due to the size, and also the need for greater mechanical strength in relation to the connector development, different printing settings were used during the slicing process, which are shown in Table 2.

**Figure 9:**
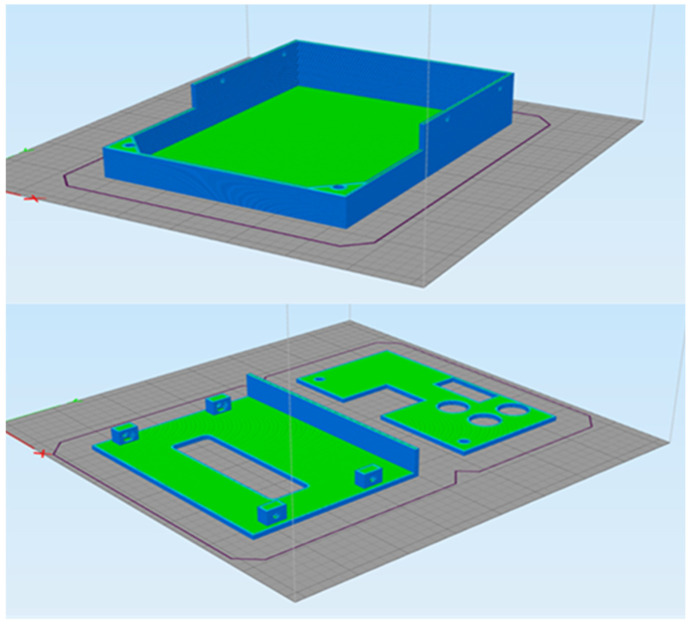
Slicing process of the electronic circuit packaging base.

Figure 9a illustrates the slicing process of the bottom of the electronic circuit’s enclosure. Table 2 shows the printing settings and Figure 9b shows the slicing process.

**Figure 10:**
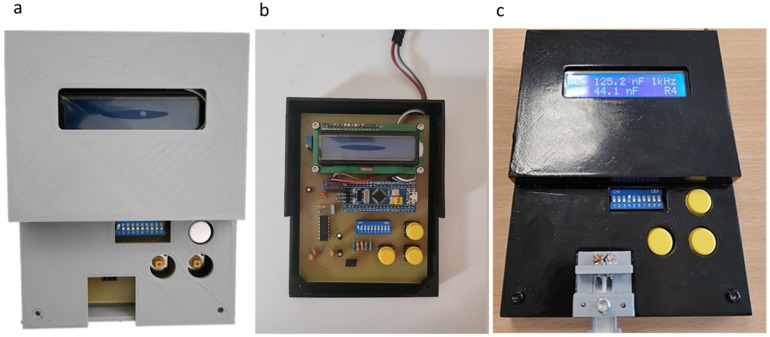
Packaging assembly: (**a**) readout circuit packaging; (**b**) the PCB placed inside the package; and (**c**) the package in its final form displaying the readings of a biosensor in its LCD screen.

All three pieces that construct the package of the measuring device are shown assembled in Figure 10a. Unlike the connector development, it was deemed necessary to further process the manufactured pieces. Specifically, a sanding and painting stage was added. Figure 10b shows the way the PCB is fixed inside the package and Figure 10c shows the final packaging of the electronic circuit, as well as where exactly the connector was placed. The LCD screen displays the reading of a biosensor.

For 3D printing, the use of a fused deposition modeling technology was selected as it is a technology accessible to the public and is not limited to narrow circles.

Although it has many advantages, such as low cost and rapid prototyping, when it comes to use in harsh environments, some disadvantages appear, such as thermal resistance. However, the results during the manufacturing of the packages were more than satisfactory. All objects were made using thermoplastic PLA, as it is an environmentally friendly material. In a different approach, it could be made using other materials such as ABS, PETG or Nylon.

## 4. Conclusions

This paper described the optimization and packaging procedure of a SARS-CoV-2 biosensor and its readout circuit. The optimization procedure involved the deposition of a photosensitive film, ORDYL SY 300, at the biosensor surface. The photosensitive film formed a layer that protects the biosensor against accidental damage. At the same time, after the exposure of the photosensitive film to UV, a well was patterned at the sensitive area of the biosensor. This well contained the liquid sample in the target sensitive area, blocking in this way its random diffusion, which can introduce a significant error factor. Two different well geometries were tested, square and circular. The circular geometry was finally selected, as in the square geometry the liquid wasn’t spreading evenly at the edges of the well. This method increased the repeatability of the measurement procedure and reduced measurement errors. In addition to the biosensor, its readout circuit was packaged as well. A protective case was 3D-printed, based on the fused deposition modeling technique. Additionally, a connector component was 3D-printed, which allowed the connection of the biosensor to the readout circuit. The optimization process improved the biosensor measurements, and the packaging allowed the use of the total detecting system for routine tests in clinics, hospitals as well as other health points of care.

## Figures and Tables

**Table 1:** Connector printing settings.

	Base	Cap and Driver	Case
Extrusion width	0.18 mm	0.2 mm	0.16 mm
Layer height	0.10 mm	0.12 mm	0.10 mm
Infill rate	30%	30%	100%
Print speed	55 mm/s	55 mm/s	55 mm/s

**Table 2:** Printing settings for circuit packaging.

	Base	Lids
Extrusion width	0.40 mm	0.26 mm
Layer height	0.30 mm	0.20 mm
Infill rate	30%	30%
Print speed	55 mm/s	55 mm/s
